# Mapping Large-Area Landscape Suitability for Honey Bees to Assess the Influence of Land-Use Change on Sustainability of National Pollination Services

**DOI:** 10.1371/journal.pone.0099268

**Published:** 2014-06-11

**Authors:** Alisa L. Gallant, Ned H. Euliss, Zac Browning

**Affiliations:** 1 United States Geological Survey, Earth Resources Observation and Science Center, Sioux Falls, South Dakota, United States of America; 2 United States Geological Survey, Northern Prairie Wildlife Research Center, Jamestown, North Dakota, United States of America; 3 Browning's Honey Company, Inc., Jamestown, North Dakota, United States of America; University of Maryland, United States of America

## Abstract

Pollination is a critical ecosystem service affected by various drivers of land-use change, such as policies and programs aimed at land resources, market values for crop commodities, local land-management decisions, and shifts in climate. The United States is the world's most active market for pollination services by honey bees, and the Northern Great Plains provide the majority of bee colonies used to meet the Nation's annual pollination needs. Legislation requiring increased production of biofuel crops, increasing commodity prices for crops of little nutritional value for bees in the Northern Great Plains, and reductions in government programs aimed at promoting land conservation are converging to alter the regional landscape in ways that challenge beekeepers to provide adequate numbers of hives for national pollination services. We developed a spatially explicit model that identifies sites with the potential to support large apiaries based on local-scale land-cover requirements for honey bees. We produced maps of potential apiary locations for North Dakota, a leading producer of honey, based on land-cover maps representing (1) an annual time series compiled from existing operational products and (2) a realistic scenario of land change. We found that existing land-cover products lack sufficient local accuracy to monitor actual changes in landscape suitability for honey bees, but our model proved informative for evaluating effects on suitability under scenarios of land change. The scenario we implemented was aligned with current drivers of land-use change in the Northern Great Plains and highlighted the importance of conservation lands in landscapes intensively and extensively managed for crops.

## Introduction

Maintaining human prosperity and quality-of-life standards requires landscapes that sustainably deliver ecosystem services valued by society. Decisions that improve the sustainability of landscapes call for information on the simultaneous responses of multiple ecosystem services to changes in land use and climate as a more comprehensive approach to safeguard against unintended, and potentially negative, outcomes of land management or policy actions [1]. Changes in land use and climate clearly alter how ecosystems function within any given landscape [2], affecting all ecosystem services, from supporting to cultural, provisioning, and regulating services [3]. Socioeconomic factors cause dynamic, often unforeseen, fluctuations and shifts in land use, so adaptive modeling systems that are quantitatively linked to monitoring activities provide both the feedback on landscape responses needed to inform decision makers and the basis for continued model improvement [2].

Socioeconomic factors are exerting considerable influence on decisions affecting land use in the Northern Great Plains of North America, a region of major agricultural production [4] and the most important area for production of continental waterfowl populations [5], [6]. The area's significance for conservation in the United States has influenced various federal, state, and nongovernmental entities to protect lands in the region from agricultural development. The U.S. Department of Agriculture administers various programs that target delivery of ecosystem services, through traditional agricultural crop programs to voluntary conservation programs. The Conservation Reserve Program (CRP) [7] is the largest of the latter and has received considerable recognition from an array of conservation interests [8], [9], [10]. The program enables landowners to enroll or re-enroll eligible farmland for 10- or 15-year contracts during specified sign-up periods or on a continuous basis for priority conservation practices, such as to support pollinators and wetlands.

Euro-American settlement of the Northern Great Plains brought conversion of vast tracts of grassland to cropland, and while grasslands continue to be converted to cropland, periods in recent decades have seen some reversal of that land-change dynamic [11], [12], [13], [14], [15]. Satellite imagery revealed a notable return of cropland to grassland in years following inception of the CRP [11], as landowners realized increased land values resulting from enrollment in the program [16]. Land conversion since the mid-2000s has favored cropland over grassland, likely influenced by the convergence of technological advances in agriculture, a push for renewable energy from biofuels, and rising commodity prices [17], [18], as well as crop insurance and disaster relief programs [12].

As incentives have risen for landowners to maximize crop acreages, Congress has reduced total allowable acreage enrollments for the CRP. The highest cap allocated for the program was 39.2 M acres (15.9 M ha; note, we report here the measurement units used in the legislation and federal reports) during 2002–2007 [19]. From 2008–2013 the cap was lowered about 20% to 32 M acres (12.9 M ha) [20]. Actual acres enrolled in CRP nationally have dropped more than 27% since the peak of the program in 2007. Reductions in enrollment in the Northern Great Plains exceeded national rates. North Dakota's peak enrollment was 338.9 K acres (137.1 K ha) in 2007, dropping 53% by 2013. The latest Farm Bill [21] specifies further, annual decrements of total allowable acres, from 27.5 M acres (11.1 M ha) in 2014 to 24 M acres (9.7 M ha) by 2017.

Concurrent interests in achieving national energy independence [22], new technology (e.g., genetically modified crops) [23], and strong economic incentives for production of agricultural crops in recent years have resulted in many private landowners converting expiring CRP acreage back into agricultural production, especially for biofuel and feedstock crops. The impact of a land-use shift of this magnitude on the ecosystem services delivered from the Northern Great Plains has been the subject of recent investigations on carbon sequestration [8], [9], wildlife habitat provisioning [10], [24], sediment reduction [9], and others. Several studies underway in the Northern Great Plains are evaluating the importance of the region for meeting the needs of national pollination services, an industry now valued at $29B (USD) annually, with about $19B attributable to honey bees [25]. No quantitative evaluations have been conducted, however, to assess how future land-use shifts may affect national pollination services.

There have been increasing concerns about declines in pollinators over past decades [26], [27], but it was the major loss of honey bees to Colony Collapse Disorder in 2007 that brought pollination services into national focus and caught the attention of the U.S. Congress and the public [28], [29]. Approximately 70% of the world's flowering plants rely on pollinators [30]. In the United States, the agricultural industry depends heavily on the pollination services of honey bees, a commercially managed insect [31], and the Nation is the world's most active market for their pollination services [32]. Numbers of managed honey bee colonies in the United States have declined by 60% since the 1940s [29], [33], while the proportion of crops in the agricultural industry that rely on their pollination services has increased [33]. In recent years rates of overwintering losses in colonies have been around 30% in the United States [34], much higher than the 18% loss rate beekeepers anticipate on average [35], and there is a concomitant perception of waning bee health [36]. Annual reports dating back to the mid-1980s also show a downward trend in national honey production [37], [38], [39], [40], [41], [42], [43], [44], [45].

A principal contributor to declines in honey bees (as well as native bees) is the loss of floral sources of pollen and nectar in the landscape [30], [46], [47]. Worldwide, conversion of land cover has been extensive, typically to support agriculture [48]. These shifts, along with trends in using synthetic fertilizers rather than rotations of nitrogen-fixing cover types (e.g., clover, alfalfa) that are beneficial to bees, challenge bees to find sufficient floral sources [30]. Understanding how land-use change affects honey bees and other pollinators is one element needed to inform decisions affecting development of sustainable management paradigms for delivering ecosystem services to society.

The Northern Great Plains carries great importance for U.S. honey production and pollination services. Commercial beekeepers from states as far away as coastal areas transport honey bees to the prairies each summer to produce honey, a crop valued at more than $256.5M nationally in 2011, with about 50% of the contribution coming from the Northern Great Plains [49]. The region typically produces most of the Nation's honey [37], [38], [39], [40], [41], [42], [43], [44], [45], but the importance of the area extends far beyond the honey crop. In 2011, over one million colonies of bees were maintained in the region during the summer, representing nearly 40% of the total number of colonies in the United States. Essentially, all of these bees are transported throughout the Nation at other times of the year to pollinate a large variety of crops, often making multiple stops in different geographic locations. Although there have been no quantitative assessments, honey bees that spend the summer in the Northern Great Plains are thought to provide 60–85% of the pollination needs for the entire Nation (Dr. Jeff Pettis, U.S. Department of Agriculture-Agricultural Research Service, personal communication). The summer locations beekeepers use for honey production in the Northern Great Plains have tremendous influence on the nutritional status and health of honey bees because foods, especially pollen, collected and stored in hives during the summer are the primary sources for nutrition during winter and other times when natural foods are unavailable. Land-cover types with abundant supplies of polyfloral and nutritious pollen are known to positively influence honey bee health through improved immune system function [50].

Although CRP lands in the Northern Great Plains have abundant polyfloral sources of pollen for pollinators, there is little information available to quantify the magnitude of that ecosystem service across large geographic extents or its contribution to national pollination services. Government policies and programs that target land resources exert influence on land-management decisions at the local level. Decisions are made parcel by parcel, but tend to accumulate to regional patterns of change [51] and can have substantial influence on ecosystem services over broad scales. Agencies that manage land resources or administer policies and programs focused on land resources seek consistent ways to evaluate outcomes of land management for ecosystem services. Ideally, these evaluations also could be conducted in a proactive context to inform decisions about policies, programs, and management actions. Within the context of developing sustainable management paradigms for landscapes, ecosystem services that are provided in one geographic location but are dependent upon conditions from other locations require special attention because they are subject to different interests and support groups [52]. Provisioning national pollination services for agricultural crops is a good example because most beekeepers are migratory and transport honey bees from pastoral locations used for honey production to distant locations for pollinating a variety of agricultural crops [53].

We describe here a prototype model we developed to quantify the influence of land use on honey bees in the Northern Great Plains to inform decisions affecting conservation programs that target delivery of national pollination services. Our objectives were to (1) develop an approach to map regional-scale habitat suitability for honey bees based on their nutritional needs from the local landscape, (2) evaluate the efficacy of available land-cover data for monitoring effects of interannual land-cover change on landscape suitability for honey bees, and (3) demonstrate how our approach can be used to assess outcomes for honey bees from drivers of land-cover and land-use change. These objectives are the foundation for a module on honey bees in a larger, integrated assessment tool developed to evaluate effects of policies, programs, climate, and other drivers of land change simultaneously across multiple ecosystem services [54].

## Materials and Methods

We prototyped our approach for North Dakota, the Northern Great Plains state with the highest production of honey among all 50 states in most years [37], [38], [39], [40], [41], [42], [43], [44], [45]. We identified local landscape criteria for honey bees and designed a spatially explicit model to locate places in the North Dakota landscape that could meet these land-cover requirements. We used available land-cover data to map interannual changes in landscape suitability, then applied our model to these maps as well as to maps we developed to fit a scenario based on recent drivers of land-use change.

### Landscape criteria for honey bees

We specified landscape criteria based on industry standards professional beekeepers use to place hives in the Northern Great Plains. These criteria consider the potential of sites to produce honey crops and to provide the nutrition needed to ensure the health of honey bees. We developed landscape criteria to support apiaries of 100 hives (Table 1, “original criteria”). This is approximately twice the number of hives a commercial beekeeper would maintain in an average apiary setting for this part of the country; however, competition for locations to place hives in the summer is high, and it is common to find multiple apiaries of 40–48 or more hives placed within overlapping forage ranges by multiple beekeepers. In many cases the cumulative stocking rate for a local landscape may exceed 100 hives.

**Table 1:** Landscape criteria used to identify potential locations for apiaries that would ensure sources of pollen and nectar throughout the growing season in the Northern Great Plains.

**Typical timing of flowering for major beneficial cover types in the Northern Great Plains**
Deciduous trees/shrubs^a^: Second half of May
Canola: First 3 weeks in June
CRP^b^ grassland and comparable mixed grass/forb: 3rd week of June through end of growing season (sometime in September)
Oilseed sunflowers: 3rd week of July through end of August
Second-crop^c^ alfalfa: Mid-July through end of growing season (sometime in September)
**Original criteria:**
≥65 ha (160 ac^d^) deciduous trees/shrubs
≥130 ha (320 ac) CRP^b^ grassland or comparable mixed grass/forb cover
≥65 ha (160 ac) alfalfa
≥65 ha (160 ac) oilseed sunflowers
≥65 ha (160 ac) canola
presence of surface water^e^
**Relaxed criteria:**
≥130 ha (320 ac) CRP^b^ grassland or comparable mixed grass/forb cover
≥130 ha (320 ac) any combination of alfalfa, oilseed sunflowers, and canola

a Occurrence of deciduous trees and shrubs in the Northern Great Plains generally is limited to windbreaks, fence lines, riparian corridors, and landscaping around houses.

b Land enrolled in the Conservation Reserve Program. Of particular interest for honey bees are grasslands rich in leguminous species because they offer long-flowering land cover with good sources of pollen and nectar.

c The first cutting of alfalfa for hay typically is done prior to blooming, but flowers are present following re-growth between subsequent cuttings.

d Acres (ac) are the unit of measure for the property system in the United States and are provided here because they relate to multiples of standard crop field sizes.

e Bees collect water for evaporative cooling in the hive, so our criteria recognize this landscape requirement in addition to sources of pollen and nectar.

### Modeling approach

Apiaries in the Northern Great Plains typically are maintained in grassland settings within proximity of roads so hives can be transported. Therefore, we first identified grassland settings sufficient in size to place commercial numbers of hives. We merged information from two sources to develop a map of grassland cover. We used proprietary data on lands with graminoid/herbaceous land treatments enrolled in the CRP [7]. These data were delineated by the Farm Service Agency with orthoimagery of high spatial resolution (typically one to several meters), and boundaries were very accurate for our type of application. For non-CRP lands we relied on grassland cover mapped from satellite data by the North Dakota Gap Analysis Program (NDGAP) [55]. The NDGAP data had lower spatial resolution (30 m) and only a fair level of mapping accuracy for grasslands (somewhat better than 50%), but provided wall-to-wall data on land cover with sub-classes that allowed us to exclude saline and sand prairie grasslands, which we deemed unlikely to provide good floral conditions for honey bees. We merged NDGAP and CRP grasslands into a single map, then fragmented the resulting grassland areas by intersecting them with the road network for North Dakota [56] to measure accessibility for transporting hives.

North Dakota requires beekeepers to register locations of their apiaries by the “quarter section” designation from the United States public land survey system [57]. We used the areal extent of a quarter section, approximately 65 ha (160 ac in the U.S. land system), as a minimum size threshold for eliminating grassland polygons that were less likely to support 100 bee colonies. We also eliminated grassland polygons that were too far from road access via information from a national gridded layer representing Euclidean distance to the nearest road [58], [59]. The road network across North Dakota largely is laid out as a grid of north-south and east-west routes, with road spacings of a mile (1620 m; although, many farms also have field access roads every half-mile or quarter-mile). We rationalized that grassland areas more than a mile from the nearest public road would be infeasible for a beekeeper to place hives; thus, we removed grassland areas having centroids (approximate centers) farther than 1620 m (1 mi) from a road. Only 0.6% of the area of the state was farther than a mile from the nearest public road, and most of this area was associated with badlands, river breaks, and large expanses of water (e.g., reservoirs). In total this criterion removed of 0.2% of the grassland centroids from further consideration as potential apiary sites. Remaining grassland polygons were at least 65 ha in size and had edges within reasonable proximity to road access. We considered the locations of centroids of these remaining polygons as potential apiary sites, though in reality an apiary could be sited anywhere within a grass polygon.

We needed to quantify acreages of desired land-cover types (per Table 1, “original criteria”) within forage distance from potential apiary sites. Forage distance for honey bees varies based on floral availability, landscape structure, and ambient conditions, such as from wind (e.g., [60], [61], [62]), but beekeepers typically look for sites providing good cover types within a distance of about 3.2 km from the hive [63], defining a forage area of 3255 ha around an apiary. We developed a separate map layer for each land-cover class of interest, determining that we needed to use a common spatial resolution of 10 m across all layers to retain certain important features (see section on “Compiling annual maps of land cover”).

**Figure 1:**
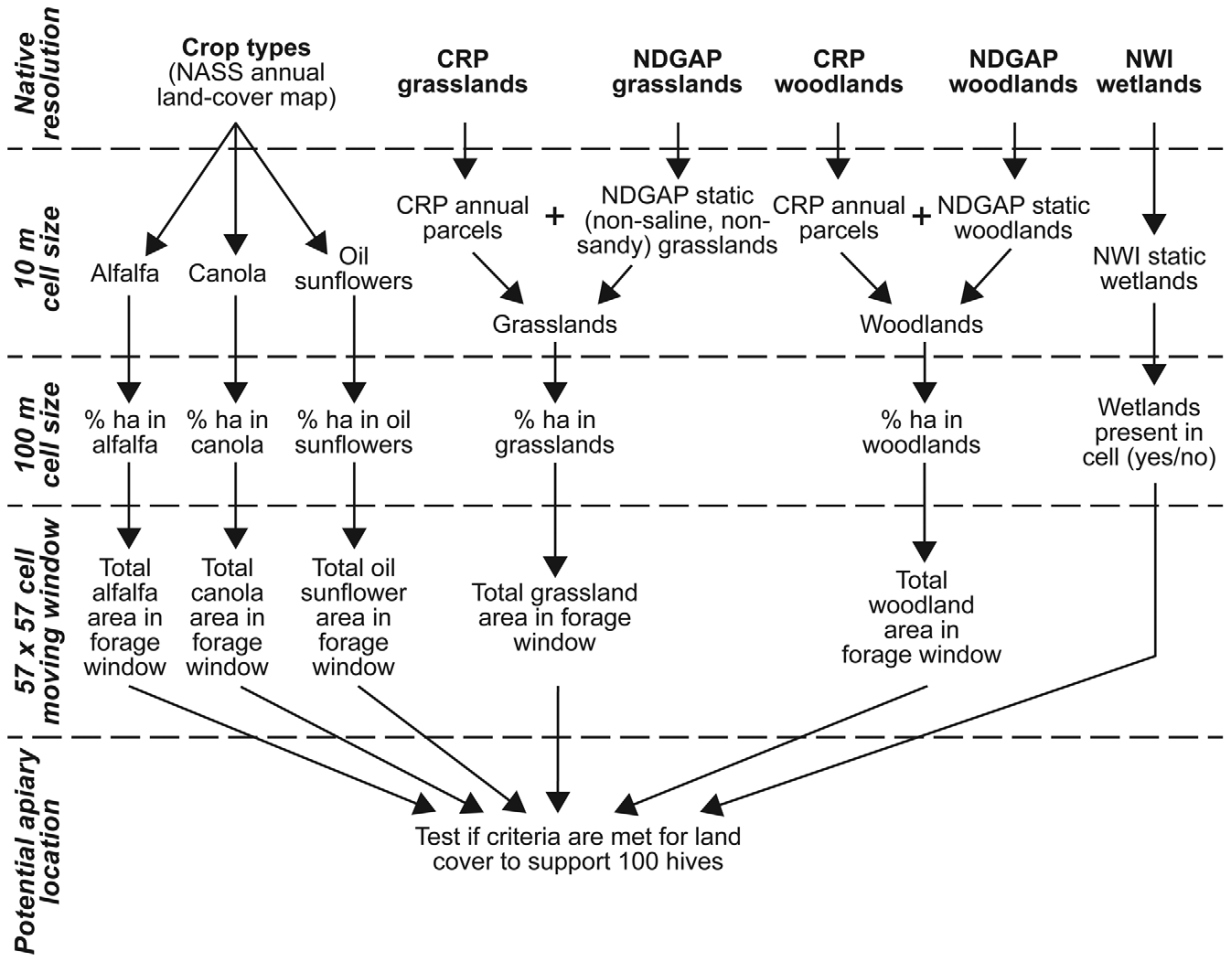
Geospatial processing schematic for compiling annual land-cover maps. We extracted land-cover components needed to meet criteria shown in Table 1 and saved them as separate gridded layers at 10-m cell resolution to retain needed spatial detail. We then summarized the proportion of area per hectare for each land-cover type (except wetlands, for which we recorded only presence/absence) and saved results as separate gridded layers at 100-m cell resolution to reduce the dimensions of the data layers. We used a moving filter of 57×57 cells to tally land-cover elements over an area comparable with the forage zone for honey bees, and queried the tallies for grassland locations we previously had identified as potential sites for placing apiaries. Figure abbreviations: NASS – National Agricultural Statistics Service, CRP – Conservation Reserve Program, NDGAP – North Dakota GAP Analysis Program, NWI – National Wetlands Inventory.

Conceptually, we wanted to move a circular forage window across North Dakota to summarize land-cover classes at each potential apiary location. This is a challenge for geospatial analytical software at the 10-m spatial resolution we used because each land-cover layer is well over 47,000 rows by 77,100 columns, and a circular forage window would encompass about 325,500 cells and require geometric calculations to determine which cells fell within the forage radius around an apiary centroid. We overcame these challenges with a modified moving-window approach (Figure 1). First, we applied a 10×10 rectangular moving window over each 10-m binary map to calculate the proportion per hectare of the respective land-cover type, then saved the results at a 100-m (one ha) cell size, yielding output layers having 0.01 times the number of cells of the original, binary layers. For example, if the original maps showed that a 10-cell×10-cell area contained 30 cells of grassland, 35 cells of non-suitable cover (such as corn and roads), 20 cells of alfalfa, and 15 cells of sunflowers, the resulting ha-sized cell would carry the information of 30% grassland, 20% alfalfa, 15% sunflowers, and 35% other (although the information for each cover type would be stored as a separate map layer, as shown in Figure 1). Second, we applied a 57×57 rectangular moving window (dimensions needed to survey acreage comparable to the forage area around an apiary) across each 100-m raster layer to calculate the amount of each cover type within forage distance from the center of the window, advancing the window one cell (100 m) at a time. We avoided the compute-intensive, circular moving window with a square window of the closest possible areal match to the size of the desired forage area. Given the imprecision of land-cover data and forage behavior by bees (influenced by wind, air temperature, and floral density, in addition to land cover type), having a round, rather than square, window was not worth the extra processing burden for a statewide application. Third, we summarized acreages across land-cover types within forage distances around all 100-m cells that had been identified as potential apiary locations to determine which sites met the areal thresholds for required land-cover types (per Table 1).

### Compiling annual maps of land cover

We compiled annual land-cover maps from multiple sources, as no single source provided all information needed to assess landscape suitability for bees. We encountered expected differences in data vintages, resolutions, and origins (see Table S1 for information sources), as well as product quality. We used the Cropland Data Layer produced annually by the National Agricultural Statistics Service (NASS) [64] for information on alfalfa (*Medicago sativa*), canola (a group of cultivars developed from *Brassica napus* and *B. campestris*), and sunflowers (*Helianthus annuus*), the three crop types needed for our analysis. All three types have been distinguished annually in map products in North Dakota since 2002, prior to which alfalfa was aggregated with grassland hay crops not nutritious for bees. We used information on woody cover types from the same two sources we used for grassland cover, the CRP and NDGAP products. The dataset for CRP-enrolled lands contained information on the years for which the conservation treatments were under contract, which enabled us to update our land-cover maps annually for both woody and grassland parcels. The NDGAP data we used for the remainder of the landscape was a single, static map derived from a collection of Landsat imagery spanning 1992–1998. We amended the NDGAP information with the relatively high-resolution wetland cover delineated by the National Wetlands Inventory (NWI) [65], as bees need sources of water and wetlands typically are poorly represented in land-cover products generated from the mid-resolution satellite data used by NDGAP [66]. The NWI map was derived from high resolution orthoimagery, but similar to the NDGAP product, represented a static depiction of wetlands derived from a collection of different orthophoto dates mosaicked across North Dakota.

The annual maps we assembled spanned 2002, the earliest year for which we could distinguish all three crop types of interest, through 2006, the latest year for which we had annual data on CRP-enrolled lands (although, we later received updated data on CRP enrollments that enabled the example application we describe in the next section). We filled the remainder of the landscape each year with data from the static NDGAP and NWI product sources. We processed all geospatial data in an equal-area map projection (following parameters used by the U.S. Geological Survey for the conterminous United States, see [67]) to maintain appropriate areal properties. All land-cover information was converted to raster layers of 10-m cell size, the spatial resolution needed to retain features from the finest-grained sources, the CRP and NWI data. We developed binary maps (”1” for presence and “0” for absence) for each land-cover type of interest, including alfalfa, canola, oilseed sunflowers, grasslands (CRP grasslands and NDGAP [non-saline and non-sandy] grasslands), wetlands, and woodlands (CRP and NDGAP woodlands).

### Assessing outcomes from a scenario of land change

We used a scenario that captured contemporary drivers of change in the Northern Great Plains to evaluate if our model could reflect influences from programs and policies on landscape suitability for honey bees. Owners of agricultural lands in North Dakota have been avid participants in the CRP. Nationwide, approximately 28 million acres (11.3 M ha) under CRP contract were set to expire between 2007 and 2010 [68]. During that same time frame, production pressures for corn-based ethanol and soybean biodiesel, the two major crops used to produce biofuels in the United States [69], [70], were on the rise [71], [72] from requirements for substantial increases in national use of renewable fuels [22]. In addition, commodity prices for corn and soybeans have been trending upwards since the mid-2000s [73]. We therefore compiled land-cover maps for 2002 and 2010 to capture conditions before and after the convergence of these pivotal influences of land-use change.

Initial results from the analysis of landscape suitability (discussed in the “Results” section) motivated us to modify some of the source information for this scenario application. We used CRP land-cover information, as before, but changed the second source of data on grasslands and woody cover from NDGAP to the 2001 National Land Cover Database (NLCD) [74]. The NLCD offered notably better mapping accuracy [75] than the NDGAP product [55] (Table S2), though did not provide information distinguishing sandy and saline grassland subtypes. For cropland information we extracted pixels from NASS maps for 2002 and 2010 across all crop types to obtain general extent of cropland for each of the years, and used specific information for canola and sunflowers to distinguish those two cover types. The accuracy for alfalfa on the NASS cropland maps was not very high; total acreages represented for alfalfa departed considerably from statistics reported from ground-based surveys conducted by NASS. Mapped alfalfa was 16x the acreage reported from ground-based surveys for 2002 and 5x the acreage reported for 2010 [76]. In addition, the distribution of alfalfa pixels tended to have a “shotgun” appearance, with many isolated pixels scattered across the landscape, rather than occurring in contiguous patches as would be expected for agricultural fields.

We developed our own maps of potential alfalfa distribution with remotely sensed data from the Landsat Enhanced Thematic Mapper sensor obtained through the Web Enabled Landsat Data (WELD) online distribution site [77], [78]. WELD data are terrain corrected and spectrally calibrated to top-of-atmosphere reflectance. We used the data layer for the Normalized Difference Vegetation Index, a data transformation that highlights photosynthetic activity in plants [79]. We targeted the WELD composite for October because it is a month in which alfalfa remains green in the Northern Great Plains after other cover types (with the exception of some grasses) have senesced for the season. For 2010 we used the WELD composite for November to fill any data gaps in the October composite. The year 2002 was exceptionally cloudy for North Dakota overpasses, so we substituted data from 2003, with the rationale that alfalfa is managed on a four- to eight–year rotation cycle in the Northern Great Plains [80], therefore minimizing potential differences in the distribution of alfalfa between 2002 and 2003. We filled data gaps in the WELD October composite for 2003 with information from the WELD Autumn composite for that year, as the November composite was contaminated with widespread clouds and snow. To generate distributions of alfalfa we simply selected thresholds of green biomass response that resulted in relatively comparable total acreages reported from the 2002 and 2010 NASS ground-based surveys. We had no means to determine the accuracy of our results, but were satisfied we had usable examples for the scenario application, given total acreage tallies and the fact our maps had much more contiguous patches of alfalfa than did the NASS cropland maps. Still, our alfalfa maps also captured grassy areas that remained green late in the year, such as along drainage courses, in other depressional areas, and in urban settings. We make no claim as to how well our land-cover compilations represented actual North Dakota land-cover patterns for 2002 and 2010 (other than the CRP data would have been fairly accurate), but our maps did provide total acreages of key land-cover types in amounts approximating those reported by NASS.

## Results

### Output from the modeling approach

**Figure 2:**
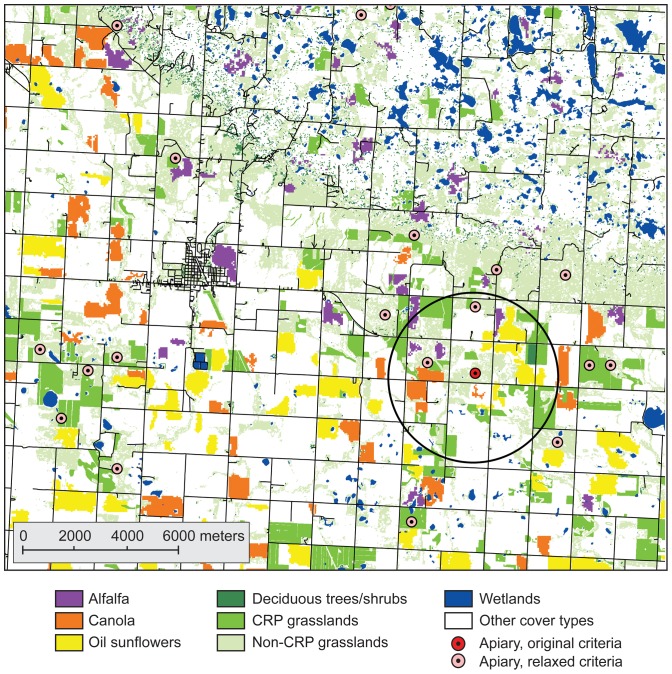
Predicted apiary locations resulting from original versus relaxed criteria for 2006. The original criteria resulted in only one apiary site identified (circle shows forage zone for this apiary), but relaxed criteria resulted in many more potential sites. Beekeepers often install apiaries in close proximity to one another in good landscapes such that forage zones are overlapping, as would be the case in this figure.

A relatively sparse number of locations met our initial land-cover criteria. We found the requirements likely were challenged by limitations in the source information we used for the annual land-cover maps. Less than half the woodlands in the landscape actually had been mapped correctly as woodlands in the NDGAP product [55], which made it hard to meet the acreage criterion for early season flowering shrubs and trees. Meeting certain crop requirements also was difficult. For example, in 2006, only 20% of the alfalfa was mapped accurately in the cropland data layer [81], meaning that 80% of the alfalfa in the actual landscape was not classified as alfalfa in the crop map. We relaxed the land-cover criteria to overcome these limitations in the source data (see Table 1, “Relaxed criteria”), then re-ran the landscape model. The model was able to find many additional locations that could meet the relaxed criteria (Figure 2), though these locations might not support apiaries as large as the 100 colonies our original criteria were defined to support.

**Figure 3:**
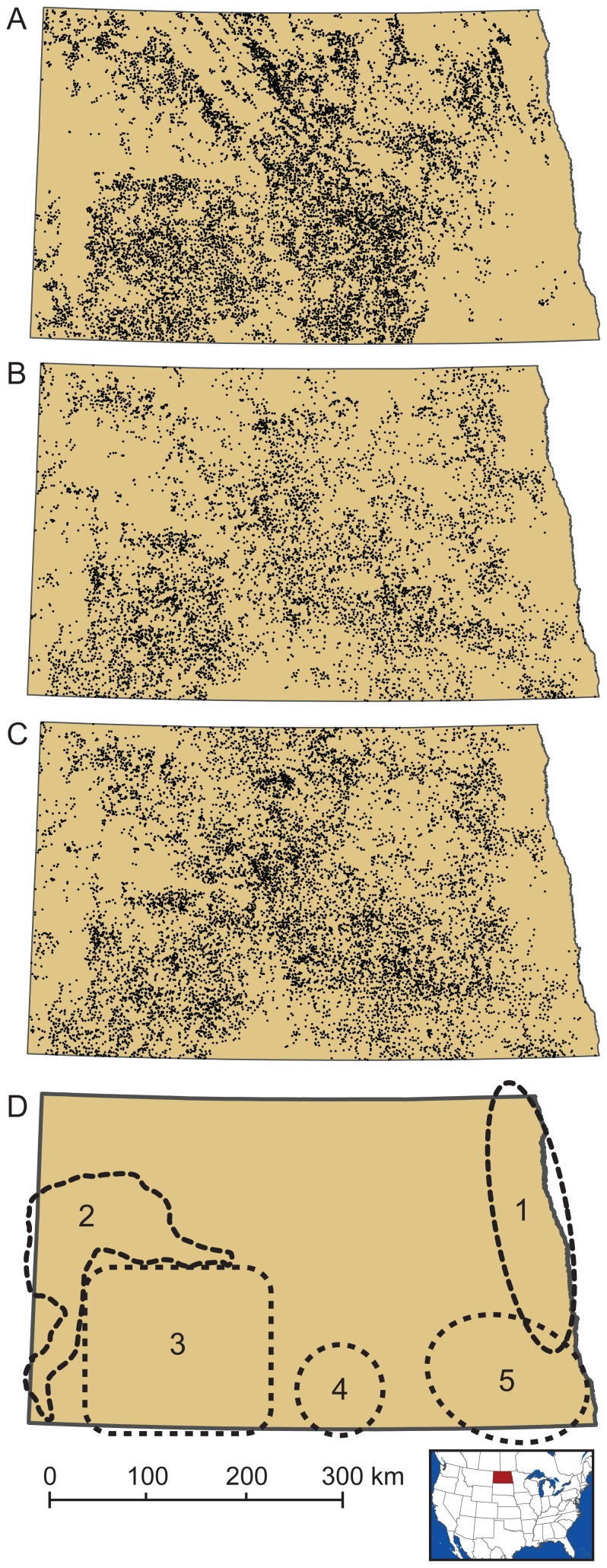
Predicted versus registered apiary locations (note: 1 dot = 1 apiary location). The cropland data we used had the highest mapping accuracies for the three crops of interest in 2002 (metadata on accuracy for all years is available through [82]). The distribution of sites we predicted to support large apiaries in 2002 (A) shares general similarities with apiaries registered for 2002 (B). Distribution of registered sites has grown more dense over time, as shown by a map of sites registered for 2010 (C). Hand-delineated areas of particular agreement (1, 2, and 3) and disagreement (4 and 5) of point distribution patterns between predicted and registered apiaries in 2002 (D), as described in the Discussion.

North Dakota requires that all apiaries be registered with the state by quarter-section legal units, so we obtained (from the North Dakota Department of Agriculture, Beekeepers Association) and mapped annual registered locations of apiaries for visual comparison with potential apiary locations produced by our model (Figure 3). We did not perform a quantitative comparison between modeled and registered sites because registered apiary sites can contain any number of beehives (based on the discretion of the beekeeper), and our model was parameterized to identify landscapes that could support large apiaries. Also, once a beekeeper registers an apiary location, they tend to re-register that same location over time, regardless if they stock it with bees, as most beekeepers do not own the property on which their hives are located and are motivated to maintain ongoing agreements with landowners. To validate this assumption we constructed a time series of registered apiary locations for North Dakota from 1981 (90 registered sites) to 2010 (10,054 registered sites). We found that the total number of sites increased every year (e.g., Figure 3B-C shows registered sites in 2002 and 2010, respectively), and rarely did any site drop from the registry. We selected 2002 to compare the distribution of locations identified with our model and registered locations for apiaries (Figure 3) because that year had the highest rates of mapping accuracy in the cropland maps for the three crop types of interest. An informal visual comparison revealed general similarities in statewide patterns of predicted locations and locations registered by beekeepers, but a notable difference is our model did not identify many locations for large apiaries in the southeastern part of North Dakota, though many apiary sites have been registered there (Figure 3D, area 5).

### Annual maps of land cover

**Figure 4:**
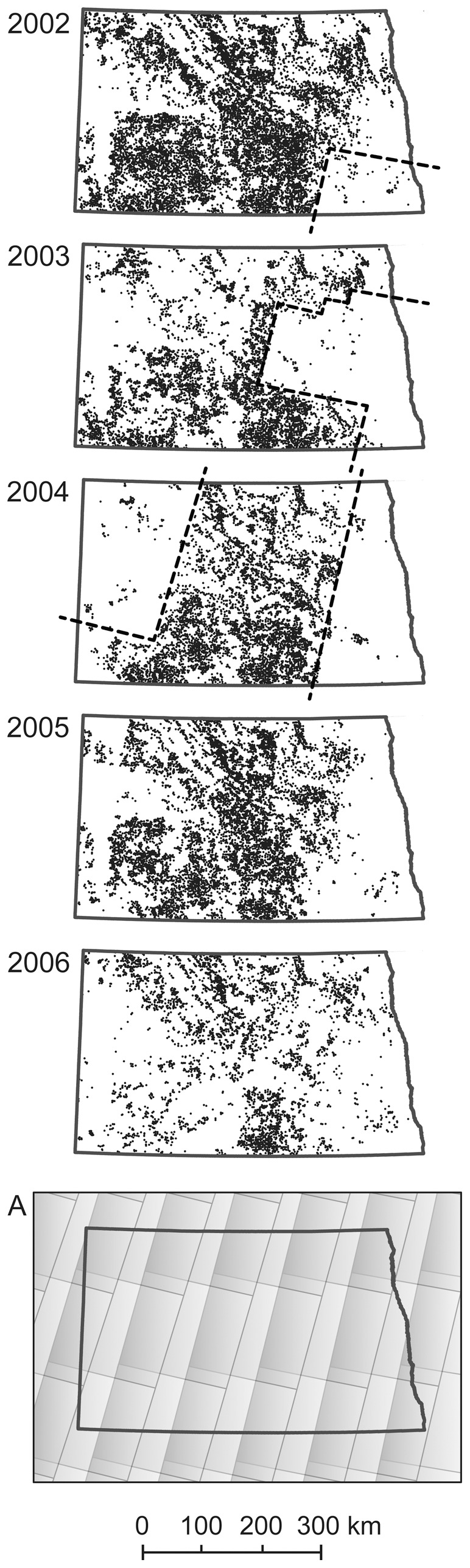
Effects of mosaicking satellite images for mapping crop types on subsequent application of resulting crop maps (note: 1 dot = 1 apiary location). Annual predicted apiary sites for 2002–2006 and Landsat scene boundaries (map A) from (side-lapping) north-south orbital paths. The influence of satellite scene boundaries on the crop-type maps derived from the set of Landsat overpasses needed to provide statewide coverage of cloud-free data are most apparent in our apiary predictions for 2002–2004 (note dashed lines). The various dates of imagery pieced together to provide statewide coverage of data often represent differences in phenological timing or other environmental conditions, making it difficult for image analysts or algorithms to detect comparable cover types across scenes [66]. This example highlights limitations of current large-area land-cover products for applications such as ours that rely on local-scale landscape components.

Our annual series predicting potential apiary locations revealed a legacy of patterns from the mosaic of Landsat imagery used by NASS to develop the crop-type maps, particularly for years 2002–2004 (Figure 4). Despite such issues associated with satellite image boundaries, many suitable locations were identified throughout the central swath of the state in all years. In contrast, we found few locations in the eastern, southeastern, and central-west parts of the state for any year (Figure 3D, areas 1, 2, and 5). Interestingly, our model consistently predicted a dense distribution of potential locations in south-central North Dakota that is not echoed by the record of registered apiaries (Figure 3D, area 4).

### Assessing outcomes from a scenario of land change

**Figure 5:**
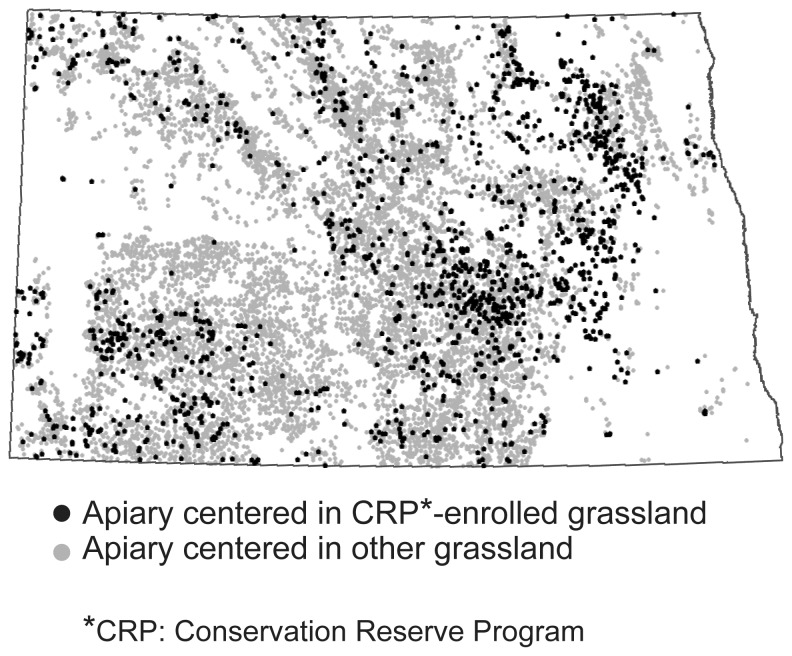
Influence of the Conservation Reserve Program (CRP). In this map we distinguish potential apiary sites that met criteria only because CRP-enrolled grasslands provided a suitable place for locating hives. This example shows how our model could be used to distinguish contributions to apiary-friendly landscapes from different sources of land management.

An important motivation to evaluate effects of land change on honey bees is to provide information on how resource managements programs and policies can influence pollination services. We highlighted predicted locations that depended on CRP-enrolled lands to meet criteria for siting apiaries (Figure 5). Landowners throughout North Dakota participate in the CRP, but the benefits for honey bees may best be realized in the eastern/southeastern part of the state, where our model found few locations outside of CRP-enrolled lands to meet the criteria for siting large apiaries.

**Figure 6:**
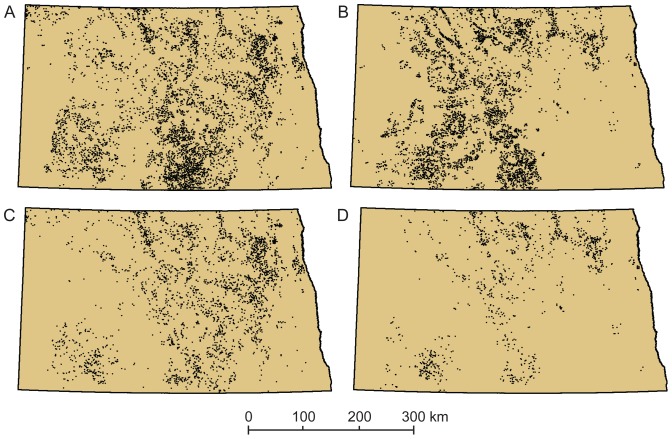
Results from a scenario of land-use change (note: 1 dot = 1 apiary location). A scenario influenced by high commodity prices and incentives to expand production of biofuel crops resulted in a loss of local landscapes suitable for apiaries. The distribution of all grassland centroids meeting criteria to support large apiaries in 2002 (A), prior to land-change incentives, is more extensive than in 2010 (B), subsequent to incentives. The same is true for the distribution of CRP centroids that met the apiary criteria in 2002 (C) versus 2010 (D).

Our results for the scenario application representing pre- and post-incentive years to expand production of biofuel crops revealed a reduction in landscape suitability for large apiaries between 2002 and 2010 (Figure 6). The model identified 10% fewer locations for large apiaries in 2010 than in 2002 across all potential grassland centroids, but the decrease in centroids located in CRP parcels was 60%. The number of CRP-enrolled polygons available as potential apiary centroids in North Dakota declined by about 15% between 2002 and 2010, but geographically this decrease represented 24% less surface area enrolled in the program in North Dakota. Coupled with changes in the surrounding land cover, much of the CRP land remaining in 2010 was unable to meet the local landscape criteria for large apiaries. This was most evident in eastern North Dakota, but substantial reduction in CRP-related sites was evident throughout most of the state.

## Discussion

Our approach to assess landscape suitability for honey bees provides the foundation for a module in a larger, integrated framework to assess multiple ecosystem services simultaneously to inform decisions on land management and policy, as well as evaluate outcomes from government conservation investments [54]. Competing drivers of change in the agricultural industry can have substantial influence on the Nation's ability to sustain the annual demand for pollination services by honey bees. For example, conservation programs supported by the Food, Conservation, and Energy Act of 2008 [20], which generally amended the conservation programs from earlier farm legislation (e.g., the CRP was in the Food Security Act of 1985 [83], have encouraged increased acreage of floral sources beneficial for bees. Conversely, the Energy Independence Security Act of 2007 [22] laid requirements for considerable increases in the volume of biofuels produced over the next decade, which currently encourages expansion of acreage in corn and soybeans, crops that provide little nutritional value for bees in the Northern Great Plains. The model we developed enables assessing outcomes from such competing influences on landscape suitability for honey bees.

### Output from the modeling approach

Informal visual comparison of the distribution of locations predicted by our model for 2002 with registered locations of apiaries for that year exhibited some general similarities (Figure 3). Both maps depict ample landscape opportunities for apiaries throughout the central swath of the state. Both maps agree the arid, erosional landscapes of the badlands and river breaks in western North Dakota and the crop types of the wall-to-wall cropland in the Lake Agassiz Plain in eastern North Dakota hold lower value for honey bees (Figure 3D, areas 1 and 2, respectively), and the maps show a strong pattern of agreement for the distribution of good apiary locations in the Missouri Plateau (Figure 3D, area 3). The maps differ most in the southeastern part of the state (Figure 3D, area 5), where our model found few locations for supporting large apiaries, yet many apiary sites are registered. It is likely these apiaries support far fewer colonies than in more floristically diverse landscapes elsewhere in the state, though this has not always been the case. According to beekeepers who have operated in southeastern North Dakota for many decades, it was rare to see corn or soybean crops in this area a decade ago, but now there is extensive cropland planted in corn, soybeans, and wheat, which are not very nutritious for bees (although, soybean varieties grown elsewhere in the United States can be beneficial for honey bees at times). In previous decades, southeastern North Dakota had many more acres of CRP, alfalfa, sunflowers, and canola (Doug Ruby, Ruby's Apiaries, personal communication). Data from the Farm Service Agency show that land enrollment in the Conservation Reserve Program for southeastern counties of North Dakota totaled 103,856 ha in 1990, rose to 163,472 ha by 2000, then dropped to 135,553 ha by 2010, and continues to drop (120,874 ha by 2013) [84]. Maps produced by the U.S. Department of Agriculture for the agricultural census series show the distribution of alfalfa has been decreasing in southeastern North Dakota since at least 1978 (though we note there is no indication canola or oilseed sunflowers were grown in much quantity in this part of the state during the past few decades [85], [86], [87], [88], [89]. Migratory beekeepers have little flexibility for shifting their operations to new regions of North Dakota to accommodate changes in land cover and instead adjust the sizes and numbers of colonies per apiary to compensate for diminished acres of forage. Given the current cropland types grown wall-to-wall in southeastern North Dakota, the benefits of land enrolled in conservation programs there can be especially important for supporting honey bees, an outcome highlighted by our model results (Figure 6). Beekeepers in that area have experienced sharp declines in honey yields as a result of these changing cropping trends, and have been required either to reduce per-site hive allotments or to abandon sites (Doug Ruby, Ruby's Apiaries, personal communication). In contrast, our model found more locations for supporting apiaries than were reflected by the distribution of registered sites in the southern part of the Prairie Coteau Slope region (see Figure 3D, area 4). The distribution of registered apiaries is not very dense in this area, but our model found plenty of locations suitable for apiaries across all years (Figure 4) because the area had many CRP-enrolled parcels, abundant acreage in oilseed sunflowers, and moderate acreages in alfalfa and mixed grass prairie.

**Figure 7:**
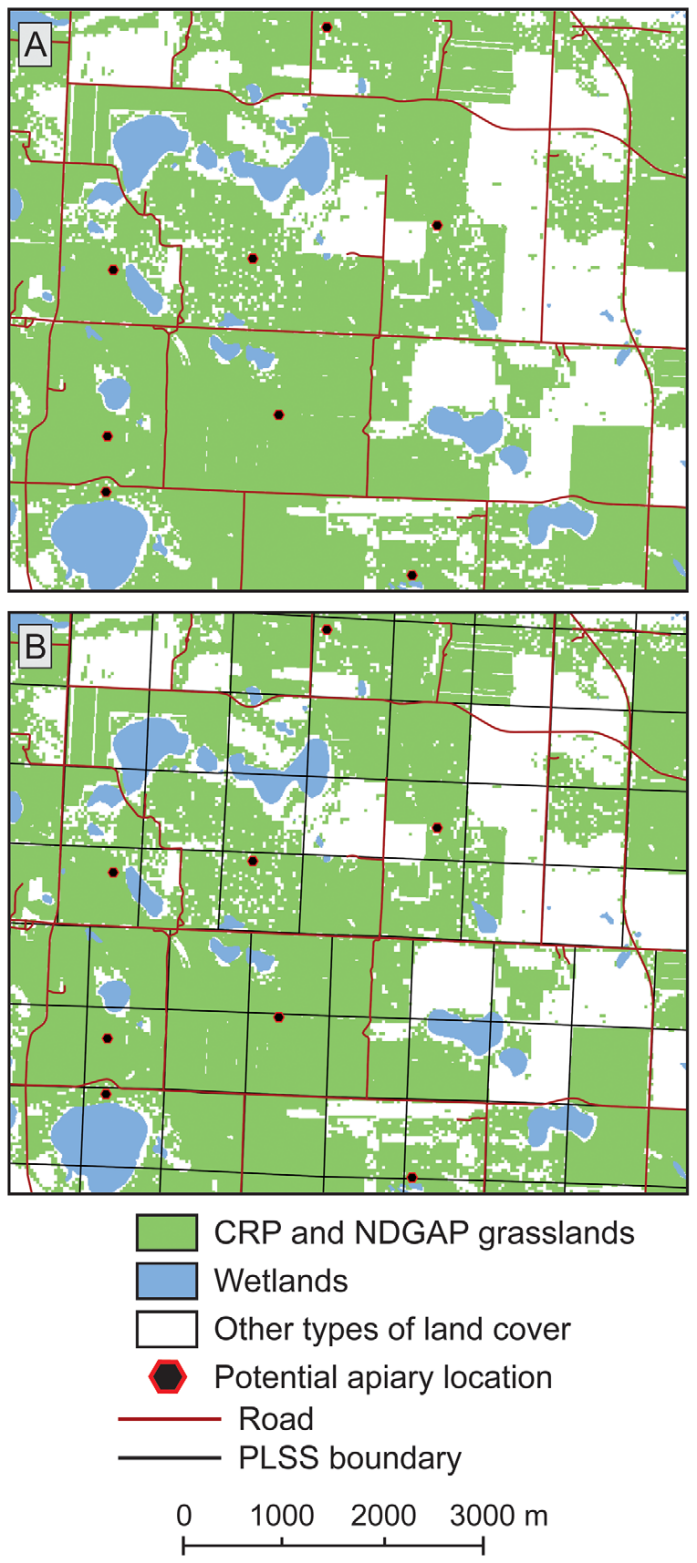
Approach we used versus future algorithm modification for identifying potential grassland sites for placing apiaries. We identified centroids (grassland locations for potential apiary sites) based on criteria for minimum extent of grassland acreage and proximity to roads (A). Density of potential site locations would increase if, instead of using grassland centroids, we applied the spatial framework of the U.S. Public Land Survey System used for registering apiary locations (B). We could maintain our same criteria for grassland extent and road access, but could have multiple apiaries within a large grassland patch, a situation that often occurs in the actual landscape. Figure abbreviations: CRP – Conservation Reserve Program, NDGAP – North Dakota GAP Analysis Program, PLSS – Public Land Survey System.

Results from our analyses helped us identify several ways to refine our model. First, the approach we developed to identify potential apiary locations was successful in implementing local landscape requirements across a statewide extent, but the all-or-nothing recipe for 100 hives is limiting. A shift from a model based completely on expert opinion to one developed statistically from data on number of hives and honey production relative to landscape setting would enable predicting suitability across a range of apiary sizes, as well as contribute information towards understanding how land-cover changes in the Northern Great Plains could affect the supply of honey bee colonies for national pollination needs. Second, we also should incorporate seasonal influences from weather into our model because of effects on plant flowering and nectar flow. Even if land-cover types were held constant across years, different weather conditions would provide substantially more or substantially less pollen and nectar because of variations in moisture and growing degree days. Third, we used centroid locations within grassland patches that met criteria for areal extent and proximity to roads to identify potential apiary sites. For future model refinement we could retain the areal and proximity requirements for grasslands, but instead use centroids from the legal mapping units (quarter sections) that beekeepers register. This would densify the number of point locations around which we could analyze land-cover components (Figure 7) and would better mimic the concentration of overlapping forage areas we have observed *in situ* with commercial apiaries.

### Using available land-cover data to monitor annual changes in landscape suitability for honey bees

The data we assembled to compile a land cover time series for North Dakota were a mix of annual and static information, and the vintages of the static sources were not always well matched to the annual sources. The Northern Great Plains have had low rates of annual land-cover conversion (typically a fraction of a percent of the total area per year) [11] during the years of our analysis and in preceding decades, providing some reassurance our assembled landscapes were relatively representative of this rather stable region of the United States. The principal land-change dynamic in the Northern Great Plains during this period was the influence of the CRP [11], for which we did have temporally dynamic data, as well as annual changes in crop types, for which we had the annual NASS data. One implicit assumption in our approach, however, was that grasslands were suitably rich in season-long floral sources. We know of no data on the floral components of land cover (except for isolated, local cases) and currently have no means to refine this assumption.

For initial trials of our model we used the NDGAP land-cover product because it offered an array of grassland and woodland subclasses that allowed us to discard those we expected to be poor for bees. The downside to using this product was relatively low rates of mapping accuracy for grassland and woodland classes. For that reason we used non-cropland land-cover information from the NLCD for our subsequent example application because NLCD had a better rate of accuracy for mapping woodland and grassland cover (Table S2) [75], [90], [91]. In addition, the NLCD provided land-cover products for 1992, 2001, and 2006 [74], which we could more easily crosswalk (than the single NDGAP product) to the vintages of our other land-cover information, and the NLCD continues to provide new national maps of land cover.

Until recently, annual mapping of land cover for the United States has been untenable at mid-resolution spatial scales. Improvements in technology and free access to Landsat data [92] have advanced this capability, as evidenced by national-scale annual products from NASS [64]. The methods employed to classify land-cover types across extensive geographic areas, however, produce maps better suited for applications that summarize outcomes at regional scales, rather than applications like ours that rely on local-scale accuracy. The results we generated based on annual land-cover maps compiled from NASS, NLCD, and NDGAP data inherited patterns of inaccuracies from those maps (e.g., Figure 4) that underscore limitations in the current state of operational development of large-area land-cover products. These maps fall short of the accuracy and precision needed for local-scale applications such as ours, an observation that has been noted elsewhere (e.g., [93]). Our algorithm relies on tallying land-cover components within a 3.2-km radius around each potential apiary site throughout the state. Local errors in individual pixels therefore can have strong influence on our results. We conclude that current operationally produced land-cover maps do not provide sufficient local accuracy for monitoring landscape suitability for honey bees. This finding underscores the need for those conducting similar habitat assessments to exercise caution in reporting shifts in landscape suitability and to evaluate if they signal mapping inaccuracies, rather than true landscape change.

There are legitimate reasons for the errors in operational-scale land-cover products. Spatiotemporal ambiguities in the spectral characteristics of land cover make it difficult to optimize a classification system for all cover types at once for all places in the landscape [66], [94], as demonstrated by image pattern effects on our annual results (Figure 4). For this reason, NASS focuses on optimizing their annual land-cover algorithms for a handful of important crops, though their maps do distinguish numerous other crop types. NASS assesses mapping accuracy for all crop types, but not for other classes of land cover. Hence, although the availability of annually updated, wall-to-wall maps of land cover is attractive for a growing population of users, the NASS maps are best employed for applications centered on the land-cover classes NASS maps with high accuracy. The metadata for the NASS cropland data layers we used (via [95]) showed the accuracy for mapping alfalfa in 2002, for example, ranged from 19% to 95% for different parts of North Dakota, and for 2004, ranged from 40% to 97%. The accuracy for mapping canola across North Dakota ranged from 88% to 97% in 2002 and from 86% to 99% in 2004. Likewise, the accuracy for mapping sunflowers varied from 80% to 99% in 2002 and 63% to 99% in 2004. The best rates of mapping accuracy at the statewide level across all years were achieved in 2002 (77% accuracy for alfalfa, 93% for canola, and 90% for sunflowers). However, even in this year when 77% percent of the land area planted in alfalfa was correctly mapped as alfalfa, the commission rate for that cover type was 55%, meaning that over half the time a pixel was called “alfalfa” it was not really alfalfa on the ground. This type of error likely contributed to the great density of locations we identified as suitable for large apiaries in 2002 compared with other years (Figure 4). We found far fewer locations that met our landscape criteria in 2006, which likely was more an artifact of a change in mapping methods used by NASS than a change in crop distribution. The NASS product for 2006 is much cleaner in appearance than in previous years, but does not provide improved mapping accuracy (alfalfa in 2006 had 80% omission error and 39% commission error; see [82]). One way NASS might improve mapping accuracy for alfalfa is to extend the range of seasonal dates of Landsat imagery they use. Alfalfa, along with some grasses, in the Northern Great Plains is one of the earliest cover types to become green in the spring, and remains green into the fall after other cover types have senesced.

### Using the model to assess outcomes for honey bees from a scenario of land-cover change

Although we found that the land-cover products currently available lack sufficient local accuracy to enable monitoring of actual landscape suitability for honey bees, our model does achieve the goal to provide a way to evaluate how different policies, programs, and market incentives could influence land cover and alter landscape suitability for honey bee colonies. Land-cover maps can be developed to meet various scenario criteria (e.g., [96]), and our model then could be applied to assess how distribution patterns of apiary locations would be affected.

The scenario we employed showed marked differences in landscape suitability prior and subsequent to agricultural and energy drivers currently influencing change in the Northern Great Plains (Figure 6). The landscapes we assembled met reported abundances of crop types important for bees in amounts comparable to those reported by NASS from ground-based sources. Ground-based statistics for 2002 and 2010 indicated decreases in acreages for alfalfa, canola, sunflowers, and CRP-enrolled land, and corresponding increases in acreages of corn and soybeans, the two major crop types associated with biofuels (Table 2) [98]. Further corroboration for shifts in land cover that affected landscape suitability is offered by the U.S. Department of Agriculture's findings that native grasslands were converted to croplands in the Northern Great Plains at a rate of about 1% (311,610 ha) per year from 1997–2007 [12], and that acreage in principal crops for North Dakota increased 117,765 ha between 2002 and 2010 [71], [99]. Also, as of 2010, North Dakota was among the top ten U.S. states for production of ethanol and biodiesel, and the amount of corn used for production of biofuels has increased sharply since 2002 [100].

**Table 2:** Comparison of acreage planted in 2002 and 2010 in North Dakota for corn and soybeans, the major crop types used to produce biofuels in the United States, versus other cover types that provide important sources of pollen and nectar for bees.

Commodity	Hectares planted in 2002 (reported acres^c^)	Hectares planted in 2010 (reported acres^c^)	Change in hectares (change in acres^c^)
Alfalfa^a^	44,515 (110,000)	32,375 (80,000)	−12,140 (−30,000)
Canola^a^	526,095 (1,300,000)	518,000 (1,280,000)	−8095 (−20,000)
Sunflowers^a^	554,420 (1,370,000)	358,150 (885,000)	−196,270 (−485,000)
CRP-enrolled lands^b^	1,346,345 (3,326,883)	1,100,510 (2,719,413)	−245,835 (−607,470)
Corn^a^	497,765 (1,230,000)	829,610 (2,050,000)	331,845 (820,000)
Soybeans^a^	1,080,515 (2,670,000)	1,659,220 (4,100,000)	578,705 (1,430,000)

a Data are from probability-based surveys of farmers conducted by the U.S. Department of Agriculture [76].

b Data are total acres enrolled, provided online by the U.S. Department of Agriculture [97].

c Acres are the unit of measure for the property system in the United States and are reported in the literature we accessed for U.S. agricultural statistics.

## Conclusions (and Future Directions)

We developed a modified moving-window approach to tally land-cover components within local-scale proximity to potential apiaries for statewide or larger regional assessments of landscape suitability for honey bees. Our work responds to a need to estimate effects from various scenarios of land change that can result from major drivers such as market values, shifts in climate, or how national programs and policies for land resources are implemented. Because the approach is sensitive to local-scale information, results based on existing land cover time series generated from remotely sensed data may reveal more about limitations in land-cover products than about actual land-cover change. Our model therefore currently is more valuable for assessing outcomes from different scenarios of land-use change than for monitoring actual changes in landscape suitability. New approaches for monitoring land-cover change with remotely sensed data are emerging (e.g., [101], [102]) that eventually will enable our model to be used as a monitoring tool in addition to a scenario-assessment tool.

Our model was parameterized based on expert opinion for a set of land-cover criteria sufficient in floral sources to support apiaries of approximately 100 hives. These criteria highlighted the best landscapes for bees in North Dakota, but is not very representative of the range of numbers of hives commercial beekeepers typically place in apiaries. Having demonstrated a method that successfully implements local-scale criteria across a large geographic extent, our future efforts will focus on developing a more sophisticated model to provide predictions covering a more realistic spectrum of apiary sizes and environmental conditions. This would be a move towards estimating the number of colonies that could be contributed from the Northern Great Plains to meet national pollination demands under different land-change scenarios.

Our motivation to develop an approach to assess landscape suitability for honey bees is to support a larger, integrated effort to evaluate multiple ecosystem services in concert. This capability is needed to provide information for land resource agencies and policy makers on potential outcomes for ecosystem services from drivers of change. Our initial efforts already have highlighted the importance of conservation lands for honey bees in landscapes extensively cropped in cover types having little pollen or nectar value for bees. Incorporating our model into the larger, integrated framework of ecosystem service models [54] will provide a more balanced assessment to support decisions related to policy, programs, and other drivers of land-use and land-cover change.

## Supporting Information

Table S1 **Information on source data.**
(DOCX)

Table S2 **Accuracy of grassland and woody land cover in maps from the National Land Cover Database and the North Dakota GAP Analysis Program.**
(DOCX)
