# Supplementary material for: Mapping Large-Area Landscape Suitability for Honey Bees to Assess the Influence of Land-Use Change on Sustainability of National Pollination Services
Source: PLoS One. 2014 Jun 11;9(6):e99268. doi: 10.1371/journal.pone.0099268 (PMC4053381; doi:10.1371/journal.pone.0099268)
Supplement: Table S1 — Information on source data. (DOCX) [file pone.0099268.s001.docx]

Supporting Table S1. Information on source data.

| Land cover information and data source | Data type/origin | Rationale for use |
| --- | --- | --- |
| Crop types: U.S. National Agricultural Statistics Service [1]. Data portal: http://nassgeodata.gmu.edu/CropScape, accessed 20 August 2012. | Gridded layer (30 m or 56 m cell size, depending on year) derived primarily from annual satellite source data (Landsat, Advanced Wide Field Sensor, and Moderate Resolution Imaging Spectroradiometer systems), along with ancillary data. | This is the only source for wall-to-wall, annual crop maps publicly available in the United States. The maps are generated from medium-resolution satellite data (ranging from 30–250 m, depending on the sensor). Accuracy of results vary by crop type, region of North Dakota, and year. |
| Deciduous trees and shrubs: Conservation Reserve Program (CRP) proprietary data [2]. | Vector layer generated from on-screen hand-delineated boundaries based on orthoimagery having a spatial resolution of one to a few meters. | Woody vegetation is sparse in the prairies, typically occurring in narrow linear features like windbreaks and riparian corridors that are difficult to detect with the spatial resolution of satellite sensors used to map U.S. land cover. We used CRP data as the most accurate and finest-resolution source of information on woodland occurrence. |
| Deciduous trees and shrubs: North Dakota Gap Analysis Program [3]. Data portal: http://gapanalysis.usgs.gov, accessed 20 August 2012. | Gridded layer developed primarily from Landsat-5 Thematic Mapper data (30 m cell size) from 1992–1998 and ancillary information from muliple sources. | There were at least three sources of information on statewide distribution of tree cover we could use for non-CRP lands. All were much coarser in spatial resolution and less accurate than the CRP data. We used the NDGAP product because it was the only source to provide the flexibility of selecting from among multiple classes of deciduous woody cover. Accuracy of land cover varies by type and region of North Dakota. |
| Grasslands: CRP proprietary data [2]. | Vector layer generated from on-screen hand-delineated boundaries based on orthoimagery having a spatial resolution of one to a few meters. | We used CRP data as the most accurate and finest-resolution source of information on grassland occurrence. The data also allowed us to highlight the contribution of this conservation program to honey bee ecosystem services. |
| Grasslands: NDGAP [3]. Data portal: http://gapanalysis.usgs.gov, accessed 20 August 2012. | Gridded layer (30 m cell size) developed primarily from Landsat-5 Thematic Mapper data from 1992–1998 and ancillary information from muliple sources. | There were at least two sources of information on statewide distribution of grasslands we could use for non-CRP lands. Both were much coarser in spatial resolution and less accurate than the CRP data. We used the NDGAP product because it was the only source to provide the flexibility of selecting from among multiple classes grasslands. |
| Grasslands and other non-cropland cover types: National Land Cover Database (NLCD; [4]). Data portal: http://www.mrlc.gov, accessed 2 February 2013. | Gridded layer developed from Landsat-5 Thematic Mapper and Landsat-7 Enhanced Thematic Mapper data (30 m cell size). Multiple vintages available. | We used NLCD data for our example scenario application after evaluating the geographic legacies of mapping errors from the NDGAP data. The NLCD products have higher mapping accuracy and more in-depth reporting of results from accuracy assessments. |
| Roads: U.S. Department of Commerce, U.S. Census Bureau [5]. Data portal: http://www.census.gov/geo/www/tiger, accessed 20 August 2012. | Vector layer from the U.S. Census Bureau Master Address File/Topologically Integrated Geographic Encoding and Referencing system. | This is the definitive national source of data on the U.S. transportation network, though it may not include the smallest, least developed roads, for example, farm access roads and other such service roads. |
| Roads: U.S. Geological Survey [6], [7]. Data portal: http://rmgsc.cr.usgs.gov/trip/data, accessed 20 August 2012. | Gridded data (30 m cell size) showing distance (km) to nearest road derived from vector layers of county road files from Geographic Data Technology, Inc. Dynamap/1000 v 8.0. | This source of information was derived from the road network data of the U.S. Census Bureau. Using this layer saved us considerable data processing to determine proximity to roads from anywhere in the landscape. |
| Wetlands: U.S. Fish and Wildlife Service National Wetlands Inventory [8]. Data portal: http://www.fws.gov/wetlands, accessed 20 August 2012. | Vector layer developed from hand-delineated boundaries on aerial photographs having a spatial resolution of one to several meters and mosaicked from dates spanning several decades. | Although this is a static representation of wetlands, it is the best data source on wetland distribution for the United States and has a mapping resolution much finer than is available from other land-cover products, which tend to miss the majority of wetlands in the Northern Plains. |
| Alfalfa: Web-enabled Landsat data [9], [10]. Data portal: http://weld.cr.usgs.gov, accessed 30 January 2013. | Gridded October 2010 composite for top-of-atmosphere data transformed to the Normalized Difference Vegetation Index (NDVI). Comparable product for November 2010 used to fill data gaps in the October output. Comparable product for October 2003 used as primary data source, with data from Autumn composite 2003 used to file data gaps (note, November 2003 composite had too much cloud and snow contamination). Developed from Landsat-7 Enhanced Thematic Mapper data (30 m cell size). | NDVI-transformed data have a long history of use for mapping primary productivity [11]. We targeted October data because few plants in the Northern Plains remain green that late in the year; these principally include alfalfa and some grasses along drainages or in landscape depressions. |

**References (the numbers used here correspond with those from the main paper)**

1. Boryan C, Yang Z, Mueller R, Craig M (2011). Monitoring US agriculture: the US Department of Agriculture, National Agricultural Statistics Service, Cropland Data Layer Program. Geocarto Int 2011: 1–18.

2. Farm Service Agency (2008) Conservation Reserve Program (CRP) benefits: water quality, soil productivity and wildlife estimates. U.S. Department of Agriculture Fact Sheet. Available: http://www.fsa.usda.gov/Internet/FSA_File/factsheet_crp_bennies.pdf. Accessed 20 July 2012.

3. Strong LL, Sklebar TH, Kermes KE (2005) A GAP analysis of North Dakota, final report. Jamestown: U.S. Geological Survey. 118 p.

4. Homer C, Fry J (2012) The National Land Cover Database. US Geological Survey Fact Sheet 2012-3020.

5. Broome FR, Godwin LS (2003) Partnering for the people: improving the U.S. Census Bureau’s MAR/TIGER database. Photogramm Eng Rem Sens 69: 1119–1123.

6. Watts RD (2005) Distance to nearest road in the conterminous United States. U.S. Geological Survey: Fact Sheet 2005-3011.

7. Watts RD, Compton RW, McCammon JH, Rich CL, Wright SM, etc. (2007) Roadless space of the conterminous United States. Science 316: 736–738.

8. Wilen BO, Bates MK (1995) The US Fish and Wildlife Service’s National Wetlands Inventory project. Vegetatio 118: 153–169.

9. US Geological Survey: WELD: Web-Enabled Landsat Data. Available: http://weld.cr.usgs.gov. Accessed 30 January 2013.

10. Roy DP, Ju J, Kline K, Scaramuzza PL, Kovalsky V, et al. (2010) Web-enabled Landsat data (WELD): Landsat ETM+ composited mosaics of the conterminous United States. Remote Sens Environ 114: 35–49.

11. Pettorelli N, Vik JO, Mysterud A, Gaillard J-M, Tucker CJ, Stenseth NC (2005) Using the satellite-derived NDVI to assess ecological responses to environmental change. Trends in Ecology and Evolution 20: 503–510.
