# Supplementary material for: Mapping Large-Area Landscape Suitability for Honey Bees to Assess the Influence of Land-Use Change on Sustainability of National Pollination Services
Source: PLoS One. 2014 Jun 11;9(6):e99268. doi: 10.1371/journal.pone.0099268 (PMC4053381; doi:10.1371/journal.pone.0099268)
Supplement: Table S2 — Accuracy of grassland and woody land cover in maps from the National Land Cover Database and the North Dakota GAP Analysis Program. (DOCX) [file pone.0099268.s002.docx]

Supporting Table S2. Accuracy of grassland and woody land cover in maps from the North Dakota GAP Analysis Program (NDGAP) and the National Land Cover Database (NLCD).

|  | **Grasslands: producer’s accuracy*** | **Grasslands: user’s**  **accuracy**** | **Woody cover: producer’s accuracy*** | **Woody cover: user’s**  **accuracy**** |
| --- | --- | --- | --- | --- |
| **NDGAP** [1] | 54% | 62% | 15% | 55% |
| **NLCD***** [2] | Region 4: 95% | Region 4: 83% | Region 4: 81% for trees, 91% for shrubs | Region 4: 90% for trees and shrubs |
|  | Region 5: 90% | Region 5: 69% | Region 5: 82% for trees, 5% for shrubs | Region 5: 88% for trees, 29% for shrubs |

*Producer’s accuracy is the proportion of reference locations (pixels) for a given class that were correctly classified. The inverse of this (100%–producer’s accuracy) is the omission error rate.

**User’s accuracy is the proportion of correctly classified locations (pixels) for a given class out of all locations labeled as belonging to that class. The inverse of this (100%–user’s accuracy) is the commission error rate.

***The conterminous United States was partitioned into 10 regions (statistical sampling strata) for conducting the accuracy assessment of the 2001 National Land Cover Database. North Dakota is part of Regions 4 and 5

**References**

1. Strong LL, Sklebar TH, Kermes KE (2005) A GAP analysis of North Dakota, final report. Jamestown: U.S. Geological Survey. 118 p.

2. Wickham JD, Stehman SV, Fry JA, Smith JH, Homer CG (2010) Thematic accuracy of the NLCD 2001 land cover for the conterminous United States. Remote Sens Environ 114: 1286–1296..
